# Peripheral Blood Mononuclear Cells Antioxidant Adaptations to Regular Physical Activity in Elderly People

**DOI:** 10.3390/nu10101555

**Published:** 2018-10-20

**Authors:** Carla Busquets-Cortés, Xavier Capó, Maria del Mar Bibiloni, Miquel Martorell, Miguel D. Ferrer, Emma Argelich, Cristina Bouzas, Sandra Carreres, Josep A. Tur, Antoni Pons, Antoni Sureda

**Affiliations:** 1 Research Group on Community Nutrition and Oxidative Stress & Laboratory of Physical Activity Science, University of Balearic Islands, 07122, Palma de Mallorca, Spain; carla_busquets@hotmail.com (C.B.-C.), xaviercapofiol@hotmail.com (X.C.); mar.bibiloni@uib.es (M.d.M.B.); miguel-david.ferrer@uib.es (M.D.F.); e.argelich@uib.cat (E.A.); cristina.bouzas@uib.es (C.B.); sandra.carreres@uib.cat (S.C.); pep.tur@uib.es (J.A.T.); antonipons@uib.es (A.P.); 2 CIBEROBN, Instituto de Salud Carlos III (ISCIII), 28029 Madrid, Spain; 3 Departamento de Nutrición y Dietética, Facultad de Farmacia, Universidad de Concepción, 4070386 Concepción, Chile; martorellpons@gmail.com

**Keywords:** active lifestyle, antioxidants, daily habits, elderly, healthy aging, mitochondria, PBMCs, ROS

## Abstract

Regular physical activity prescription is a key point for healthy aging and chronic disease management and prevention. Our aim was to evaluate the antioxidant defense system and the mitochondrial status in peripheral blood mononuclear cells (PBMCs) and the level of oxidative damage in plasma in active, intermediate and inactive elderly. In total, 127 healthy men and women >55 years old participated in the study and were classified according on their level of declared physical activity. A more active lifestyle was accompanied by lower weight, fat mass and body mass index when compared to a more sedentary life-style. Active participants exhibited lower circulating PBMCs than inactive peers. Participants who reported higher levels of exercise had increased antioxidant protein levels when compared to more sedentary partakers. Carbonylated protein levels exhibited similar behavior, accompanied by a significant raise in expression of cytochrome c oxidase subunit IV in PBMCs. No significant changes were found in the activities of antioxidant enzymes and in the expression of structural (MitND5) and mitochondrial dynamic-related (PGC1α and Mitofusins1/2.) proteins. Active lifestyle and daily activities exert beneficial effects on body composition and it enhances the antioxidant defenses and oxidative metabolism capabilities in PBMCs from healthy elderly.

## 1. Introduction

Aging is a normal and multi-factorial physiological phenomenon characterized by a progressive generalized deterioration of the different functions in the organism resulting in an increased vulnerability to environmental challenge and a growing risk of disease and death [1]. This decline of homeostatic capacity is also associated with a general decrease in the effectiveness of mechanisms involved in the damage prevention or reparation [2,3]. One of the main problems associated to aging is the appearance of numerous pathological conditions, almost all non-communicable disorders, such as diabetes, cardiovascular diseases, neurodegenerative disorders or various types of cancer. Altogether, with the increase in life expectancy, the socio-health cost associated to aging increases notably and becomes a problem for the countries. In this sense, it is essential to develop strategies aimed to achieve a healthy aging, improve the quality of life of the elderly and reduce the economic cost associated to aging [4].

It is well reported in the literature that physically active lifestyles promote health and healthy aging [5,6,7,8]. The beneficial effects of exercise on various physiological and psychological parameters in the elderly have also been well established [9,10]. The metabolic challenge during the performance of physical activities results in an elevated generation of reactive oxygen species (ROS), produced as normal cellular products of metabolism [11,12]. ROS produced at moderate levels are influential modulators of muscle contraction, generate physiological responses and, although it may seem contradictory, exert antioxidant protection of the organism [13,14,15]. This fact brings to light that the intensity, duration, and mode of physical activity markedly affect the metabolic and molecular response to a given exercise challenge [6].

Multiple works focus on skeletal muscle biopsies because it is a highly malleable tissue, capable of pronounced metabolic and morphological adaptations in response to contractile activity (i.e., exercise) [16]. However, the assessment of metabolic function in cells isolated from human blood for treatment and diagnosis of disease is a new and important area of translational research. It is now becoming clear that some diseases also modulate mitochondrial energetics in platelets and leukocytes. This opens the possibility that these circulating cells could sense metabolic stress in patients and serve as biomarkers [17,18] of mitochondrial dysfunction in human pathologies such as diabetes, neurodegeneration and cardiovascular disease [19]. The minor invasiveness, reliability and speed in extraction and purification of peripheral blood mononuclear cells (PBMCs) and neutrophils constitute an emerging approach for using blood cells as an in vivo and in vitro tool to assess the impact of diverse stressors on physiological parameters such as antioxidant and mitochondrial status. The potential of isolated PBMCs to determine antioxidant status and mitochondrial status has been previously described. It has been evidenced that acute exercise enhances mitochondrial biosynthesis, fission and fusion processes in PBMCs [20,21,22] and training stimulates not only the biogenesis of mitochondria but also the removal of old and unhealthy mitochondria through mitochondrial dynamics and autophagy in skeletal muscle [23]. Conversely, other studies have assessed the antioxidant status in lymphocytes, included in PBMCs [24,25,26,27]. In addition, dysregulated nuclear factor kappaB (NF-κB) pathway in PBMCs and increased oxidative stress in mitochondria isolated from lymphocytes were found in subjects with mild cognitive impairment and may potentially reflect the brain damage [28,29].

To sum up, the expected increase of the elderly population is an important health challenge in our society, and maintaining an active lifestyle is also a key point for chronic disease management and prevention. The aim of the present study was to evaluate the antioxidant defense system and the mitochondrial status PBMCs of elderly persons, as well as to assess the oxidative damage in plasma, and its association with different degrees and frequency of daily activities.

## 2. Materials and Methods

### 2.1. Study Population, Experimental Design and Ethics

This study is encompassed within a cross-sectional study aimed at identifying cardiovascular risk factors in elderly population. The inclusion criteria were men 55–80 and women 60–80 years old with no previously documented cardiovascular disease (stroke, ischemic heart disease, angina, and myocardial infarction) and Diabetes Mellitus type 2 (patients treated with insulin/oral hypoglycemic, basal blood glucose > 126 mg/dL, or casual glycemia > 200 mg/dL with symptoms of diabetes or Glucose Oral Tolerance Test with glycemia > 200 mg/dL in two determinations) or that meet three or more of the following factors: (a) smoking (smokers of more than one cigarette a day or smokers who have stopped smoking in the last year); (b) hypertension (subjects with arterial pressures ≥ 140/90 mm Hg without treatment or those who follow hypotensive treatment regardless of their tensional numbers); (c) hypercholesterolemia (subjects with LDL-cholesterol > 160 mg/dL without treatment or those who follow a hypolipemiant treatment regardless of your LDL-cholesterol levels); (d) HDL-cholesterol < 40 mg/dL, with or without lipid-lowering treatment; (e) overweight or obesity (body mass index > 25 kg/m^2^); and (f) family history of early ischemic heart disease (first-class relatives men <55 years old or women <65 years old).

Exclusion criteria: all those subjects that do not meet the protocol requirements or who have any of the following criteria have been excluded: (a) institutionalized patients, who do not live independently or cannot stand up; (b) patients without fixed residence in the last years or with the impossibility to attend the quarterly controls; (c) patients with acute inflammatory pathology (e.g., pneumonia) may participate in the study after three months of healing; (d) body mass index > 35 kg/m^2^; (e) immunosupressed or HIV-infected patients; (f) chronic alcoholics or drug addicts; and (g) patients who have received drugs under investigation during the last year; (h) Illiteracy.

A total sample of 127 participants (61 men and 66 women) met the requirements to be included in the study. The participants were asked to respond questionnaires of nutritional habits, lifestyles and physical activity. The physical activity performed by the subjects was measured using the Minnesota Leisure-Time Physical Activity Questionnaire which was validated for the Spanish old adult population [30,31]. This questionnaire included a list of physical activities and the participants were asked about what type of leisure-time physical activities (LTPA) they had performed during the previous year. To avoid memory bias, as far as possible, the marked activities performed during the last week were collected first, and, in turn, those performed the last month, last quarter, and finally the last year, always including the former periods. For validation purposes, only the information referring to the last year was used. Thus, this questionnaire does not examine the last exercise performed, but rather the frequency and type of activity within the last week/month/year. The metabolic equivalents (METs) corresponding to the activities on the list was defined according to current knowledge [32]. The participants estimated the duration of the activities performed in min/week, and then, the participants were classified according the METsmin/week. Interviewers took basic anthropometric measurements and nurses took blood samples at the health center closest to the address of the respondent.

The study was conducted according to the guidelines laid down in the Declaration of Helsinki and all procedures were approved by the Ethics Committee of Research of Balearic Islands (CEIC-IB1295/09 PI). All participants were informed of the purpose and the implications of the study, and informed consent was obtained from all subjects.

### 2.2. Body Composition and Dietary Intake

Anthropometric measurements were performed by professional observers to minimize the inter-observer coefficients of variation. Height was determined using a mobile anthropometer (Seca 213, SECA Deutchland, Hamburg, Germany) to the nearest millimeter, with the subject’s head in the Frankfurt plane. Body weight, body fat and muscle mass were determined using a Segmental Body Composition Analyzer according to manufacturer’s protocol (Tanita BC-418, Tanita, Tokyo, Japan). To avoid variability in the values, the measurement was taken after 12 h of rest and subjects refrained from excessive eating and drinking the day before the analysis. The participants were weighed in bare feet and light clothes, and subtracting 0.6 kg for their clothes. Weight and height measures were used to calculate body mass index (BMI, kg/m^2^). Dietary habits were assessed using the 24 h dietary recall method, a structured interview intended to track detailed information about all foods and beverages consumed by the respondent in the past 24 h, from midnight to midnight of the previous day [33,34]. A dietician team verified and quantified the food records and every food item consumed was converted into nutrients using a computerized program according to the European and Spanish food composition tables.

### 2.3. Cell Isolation and Cell Viability Test

Venous blood samples were collected under basal conditions from the antecubital vein of participants in the study in suitable vacutainers with EDTA as anticoagulant in overnight fasting conditions. The PBMC fraction was purified from fresh whole blood and isolated following a protocol described previously [35] using Ficoll–Paque PLUS reagent (GE Healthcare, Chalfont St Giles, UK) [36,37]. Briefly, 6 mL of blood was carefully introduced on 4 mL of Ficoll (proportion of 1.5:1) and was then centrifuged at 900× *g*, at 4 °C for 30 min. The plasma and the Ficoll phases were discarded and PBMCs layer was washed twice with phosphate-buffered saline (PBS), pH 7.4, and centrifuged for 10 min at 1000× *g*, 4 °C. Plasma was obtained by centrifuging whole fresh blood at 1700× *g* for 15 min, 4 °C.

### 2.4. RNA Extraction and Real-Time PCR

Cytochrome c oxidase subunit IV (COXIV), Peroxisome Proliferator-Activated Receptor Gamma Coactivator (PGC-1α), Mitochondrial NADH Dehydrogenase Subunit 5 (MitND5) and Mitofusins1 and -2 (Mtf1/2) mRNA expression was determined by Real-Time PCR based on incorporation of a fluorescent reporter dye and using human 18S ribosomal as the reference gene. For this purpose, total RNA was isolated from PBMCs by extraction with Tripure^®^ (Tripure Isolation Reagent, Roche Diagnostics, Mannheim, Germany) following a procedure previously described (Capó et al., 2014). RNA (1 μg) from each sample was subjected to reverse transcription using 50 units of Expand Reverse Transcriptase (Roche Diagnostics, Germany) and 20 pmol of oligo (dT) for 60 min at 37 °C in a 10 μL final volume, according to the manufacturer’s instructions. The resulting cDNA (3 μL) was amplified with the Light Cycler FastStart DNA MasterPLUS SYBR Green I kit (Roche Diagnostics, Germany). Target cDNAs were amplified as follows: 10 min, 95 °C followed by 45 cycles of amplification. The specific primers and amplification conditions used for each gene are presented in Table 1 mRNA levels from inactive women were arbitrarily referred to as 1.

### 2.5. Enzymatic Determinations

Catalase (CAT) activity in plasma and PBMCs was measured by the spectrophotometric method of Aebi [38]. Superoxide dismutase (SOD) activity was measured in plasma and PBMCs by an adaptation of the method of McCord and Fridovich [39]. Glutathione reductase (GRd) activity was measured in PBMCs by a modification of the Goldberg and Spooner spectrophotometric method [40]. Glutathione peroxidase (GPx) activity was determined using an adaptation of the spectrophotometric method of Flohé and Gunzler [41]. All activities were estimated in PBMCs and/or plasma samples with a Shimadzu UV-2100 spectrophotometer (Shimadzu Corporation, Kyoto, Japan) at 37 °C.

### 2.6. SDS-Polyacrylamide Gel Electrophoresis and Western Blot Analysis

Antioxidant protein levels in PBMCs were determined by Western blot analysis. Twenty-microgram protein aliquots were loaded in each lane of an sodium dodecyl sulfate (SDS) polyacrylamide gel (15% acrylamide) and electrophoresed by molecular weight at 200 V for 90 min. Bands were electrotransferred onto a nitrocellulose membrane by using Trans-Blot^®^ Turbo™ Transfer System (Bio-Rad, Segrate, Milan, Italy). The membrane was blocked (5% non-fat powdered milk in PBS, pH 7.5, containing 0.1% Tween 20) for 5 h and incubated with the corresponding primary monoclonal antibody. Antibodies anti-catalase (CAT) (1:1000, rabbit), Mn superoxide dismutase (MnSOD) (1:1000, mouse), glutathione reductase (GRd) (1:1000, mouse), glutathione peroxidase (GPx) (1:200, mouse), thioredoxin reductase 1 (TrxR1) (1:200, goat) and uncoupling protein 3 (UCP3) (1:500, mouse) were supplied by Santa Cruz Biotechnology (Santa Cruz, CA, USA). Blots were then incubated with a secondary peroxidase-conjugated antibody (1:10,000) against specific primary antibody. Development of immunoblots was performed using an enhanced chemiluminescence kit (Immun-Star^®^ Western C^®^ Kit reagent, Bio-Rad Laboratories, Hercules, CA, USA). Protein bands were visualized using the image analysis program Quantity One (Bio-Rad). Precision Plus Protein Kaleidoscope™ (Bio-Rad) was used as a molecular weight marker.

### 2.7. Malondialdehyde Assay

Malondialdehyde (MDA) concentration (µM), as a marker of lipid peroxidation, was analyzed in plasma by a colorimetric assay kit (Sigma-Aldrich Merck^®^, St. Louis, MO, USA) following the manufacturer’s instructions. The method used is specific for MDA determination and is based on the reaction of MDA with a chromogenic reagent to yield a stable chromophore with maximal absorbance at 586 nm. Briefly, plasma samples or standards were placed in glass tubes containing n-methyl-2-phenylindole (10.3 mM) in acetonitrile: methanol (3:1). HCl 12N was added and the samples were incubated for 1 h at 45 °C. The absorbance was measured at 586 nm. MDA concentration was calculated using a standard curve of known concentration.

### 2.8. Protein Carbonyls and Nitrotyrosine Determination

Protein carbonyl derivatives and nitrotyrosine (Nitro-Tyr) levels were determined in PBMCs by an immunological method using the OxyBlotTM Protein Oxidation Detection Kit (Chemicon International Inc, Katy, TX, USA) following the manufacturer’s instructions. Total protein concentrations were measured by the method of Bradford [42]. Initially, samples (10 µg or 150 µg of protein for carbonyl or nitrotyrosine, respectively) were transferred onto a nitrocellulose membrane by the method of dot-blot using a Bio-Dot^®^ Microfiltration Apparatus (Bio-Rad, Segrate, Milan, Italy). For carbonyl determination, the membrane was incubated in the presence of 2,4-dinitrophenylhydrazine (DNPH) after transference. Once derivatized, the membrane was incubated with the primary antibody, specific to DNP moiety proteins in the case of carbonyl determination or rabbit anti-nitrotyrosine antibody for nitrotyrosine determination. This step was followed by incubation with horseradish peroxidase-antibody (goat anti-rabbit IgG) conjugate directed against the primary antibody. The membrane was then treated with luminol, which is converted into a light-emitting form at wavelength 428 nm by the antigen/primary-antibody/secondary-antibody/peroxidase complex. The light was visualized and detected by short exposure to a Molecular Imager Chemidoc XRS (Bio-Rad, Segrate, Milan, Italy). Image analysis was performed using Quantity One-1D analysis software (Bio-Rad, Segrate, Milan, Italy).

### 2.9. Statistical Analysis

Statistical analysis was carried out using the Statistical Package for Social Sciences (SPSS v.21 for Windows, IBM Software Group, Chicago, IL, USA). Results are expressed as mean ± standard error of the mean (SEM), and the level of significance was established at *p* < 0.05 for all statistics. Normality of data was assessed using Kolmogorov–Smirnov test. The statistical significance of the data was checked by two-way analysis of variance (ANOVA) after adjustment for gender (G) and exercise (E). When significant differences were found between groups, a Bonferroni post hoc test was carried out. Inactive women were taken as a reference group and referred to as 1.

### 2.10. Limitations of the Study

Firstly, the present cross-sectional design gives limited ability to elucidate causal relationship between exercise and antioxidant adaptations. Secondly, physical activity was not measured objectively such as by using accelerometer. Thirdly, the macronutrient intake was estimated using recall diets instead of food frequency questionnaires that have been questioned in epidemiological studies [43,44]. Two 24-h recall diets tend to underestimate the food intake over a large period compared to food frequency questionnaires, and imply a considerable day-to-day variation in macronutrient intake. Fourthly, underreporting was calculated using energy intake/basal metabolic rate and medications types (e.g., antidepressants, influence weight, etc.) that might influence basal metabolic rate and lifestyle factors as physical activity were not considered in the present study.

## 3. Results

### 3.1. Anthropometric Parameters and Dietary Intake

Participants in the study were separated by gender and classified into three groups (active, intermediate and inactive) in accordance to the physical activity performed (Table 2). The variables measured in this study were age, weight, height, body mass index (BMI), body fat percent, and metabolic equivalents (METS, defined as the amount of oxygen consumed while sitting at rest, and is equal to 3.5 mL O_2_·kg^−1^·min^−1^) [45]. Significant differences in weight and height were found between men and women: men were heavier and taller than women and had higher values of body fat. Women exhibited similar weight, height, without significant differences in any of the anthropometric parameters depending on the degree of physical activity except for BMI: inactive women exhibited a higher percentage of body fat than the intermediate and active groups. Men constituted a more heterogeneous group and some significant differences were found between groups depending on the degree of physical activity. Active men had statistically significant lower weight, body fat and BMI when compared to the inactive peers. The degree of physical activity evidenced a progressive and significant increase in the calculated METs in both women and men.

Energy, macronutrient and micronutrient intake of the participants in the study are presented in Table 3. No significant differences were observed in macronutrient, vitamins C and E, selenium and zinc dietary intake between active, intermediate and inactive groups. Daily selenium and zinc intakes were significantly higher in men than women. Energy intakes ranged 983–2996 kcal/day for women and 823–3348 kcal/day for men. Under-reporters (energy intake/basal metabolic rate < 0.96) were 33.8% (17 women and 26 men) [46]. However, when macronutrient intake was compared between under-reporters and non-under-reporters, no statistical significant differences were found except for daily fiber intake (significances not shown). Thus, under-reporters were not excluded from the present analysis.

PBMC blood count and the percent of lymphocytes and monocytes are shown in Table 4. Statistically significant changes in PBMCs absolute counts were found in active groups. Both women and men who reported higher levels of exercises practiced on a regular basis exhibited lower cell count than intermediate and inactive subjects.

### 3.2. Antioxidant Protein Levels and Oxidative Stress Markers

The levels of the antioxidant proteins CAT, MnSOD, GRd, GPx, TrxR1 and UCP3 in PBMCs were evaluated in inactive, intermediate and active men and women in basal conditions (Table 5). Except for MnSOD in men and UCP3 in women, the rest of the proteins studied exhibited statistically significant higher values in active participants than inactive subjects, taking inactive women as a reference group. In addition, TrxR1 levels were significantly higher in intermediate men than inactive men. Significant differences between active and intermediate were also found for GRd in women and UCP3 in men. Gender differences were only reported in CAT and GRd in the intermediate group with higher values in men. No significant interactions between gender and exercises were found.

**Figure 1:**
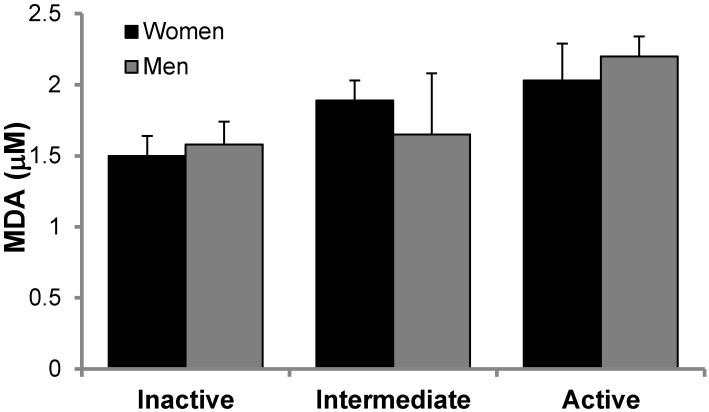
MDA levels in plasma men and women in the study, classified according to physical activity. No significant effects of gender or exercises were found. Statistics: Two-way ANOVA. Results are presented as Mean ± SEM.

**Figure 2:**
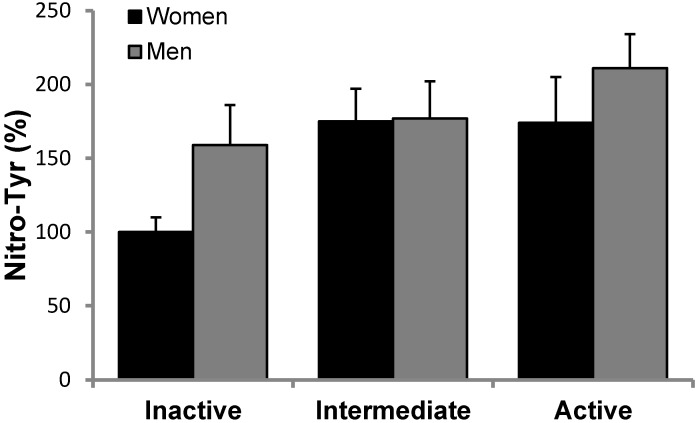
Nitro-Tyr levels in PBMCs men and women in the study, classified according to physical activity. No significant effects of gender or exercises were found. Statistics: Two-way ANOVA. Results are presented as Mean ± SEM.

**Figure 3:**
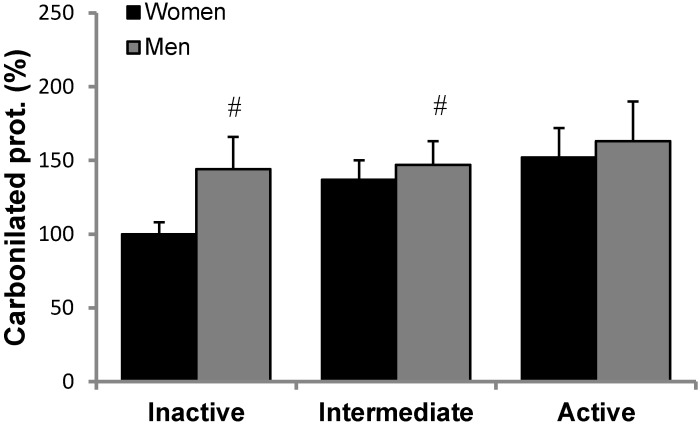
Carbonylated protein levels in PBMCs men and women in the study, classified according to physical activity. Statistics: Two-way ANOVA. Results are presented as Mean ± SEM, *p* < 0.05. E indicates significant effects of exercise. # indicate differences with respect to the inactive group.

Oxidative stress markers are reported in Figure 1. MDA levels (Figure 1) were measured in plasma, whereas Nitro-Tyr (Figure 2) and carbonylated proteins (Figure 3) were measured in PBMCs of inactive, intermediate and active men and women. Although there is a trend in MDA and Nitro-Tyr to increase with exercise, the differences were not statistically significant. Carbonylated proteins in PBMCs reported significant higher levels in the intermediate and active groups than the inactive group but only in women.

### 3.3. mRNA Relative Expression and Enzymatic Activity

COXIV, PGC1α, MitND5, Mtf1 and Mtf2 relative mRNA expression was assessed in PBMCs. Results are found in Table 6. The mRNA expression of COXIV significantly increased five- and four-fold in active women and men, respectively, compared to the control group (inactive women.). PGC1α, MitND5 and Mtf1/2 exhibited some fluctuations in their mRNA expression depending on the degree of physical activity of the group, but none of these changes were statistically significant.

Results of enzymatic activities of CAT and SOD in plasma and CAT, SOD, GPx and GRD in PBMCS are shown in Table 7.

## 4. Discussion

The present study attempts to assess the antioxidant system and the mitochondrial status in PBMCs and the level of oxidative damage in plasma, in the elderly according to different degree of physical activity. The changes observed in antioxidant markers in PBMCs cannot be attributed to dissimilar ingests since no significant differences in macro- and micronutrients were found among active, intermediate and inactive groups. The main finding of the present study is the existence of a direct relationship between the self-reported physical activity and protein levels of enzymatic antioxidants and mitochondrial oxidative capacity in both men and women. Men who reported a more active lifestyle exhibited significantly lower weight, BMI and body fat. Women only exhibited significant changes in body fat: it was lower in those who reported more daily activities. The association among body fat, inflammatory markers, and oxidative stress has been previously investigated [47,48,49]. The adipose tissue is a complex organ with functions other than energy storage, including secretion of several adipokines, such as TNF-α, IL-6, CRP and resistin, among others. Indeed, it has been proposed that adipose tissue may be a significant contributor to increased systemic inflammation in overweight and obese subjects, which can positively correlate to augmented oxidative stress [48]. More active subjects in our study exhibited significant lower BMI and body fat, a fact that would contribute to the attenuated inflammatory status in these elderly participants (observed by the lower circulating PBMCs) and, consequently, enhanced antioxidant machinery (observed by higher antioxidant protein levels). The low number of PBMCs in the active group could derive from the presence of lower amounts of effector memory T cells, exhibiting a reduced proliferative capacity and shorter lifespan, which has been reported to be significantly reduced in a trained group compared with a non-trained group [50].

The significantly higher levels of antioxidant proteins in active participants than inactive partakers match the statement that frequent exposure to moderate exercise (chronic stress) may exert protective effects against oxidative damage. This fact is largely attributed to the upregulation of endogenous antioxidant enzymes [51] such as mitochondrial MnSOD, GPx, and CAT among others [52,53]. These results would be in accordance with previous investigations reporting that antioxidant defenses are enhanced in trained subjects [54,55,56]. Thus, the exposure of cells to regular daily activities and an active lifestyle leads to a scenario in which the antioxidant defenses are activated and ready to cope with subsequent bouts of activity and there are no significant raises in oxidative damage markers in PBMCs.

Even though PBMCs from participants who reported regular physical activity exhibited increased antioxidant protein levels, active men and women did not show significant changes in their antioxidant enzymes activities in both plasma and PBMCs. This discrepancy between protein levels and observed activities may be due to the ability of antioxidant enzymes to be induced in the face of a stressful situation, such as exercise, to adapt quickly to the new demanding situation. In this sense, a previous study showed that performing a duathlon in healthy athletes is able to increase enzyme activity, but without altering protein levels suggesting a greater degree of activation of the enzymes present [57]. This fact was later evidenced in in vitro studies, where it was observed how ROS itself at low/moderate levels can activate antioxidant enzymes. The CAT activity of hemolyzed erythrocytes was increased when subjected to xanthine/xanthine-oxidase-generating superoxide anion system [57], while an increase in superoxide anion levels directly activates SOD activity [58]. In another study, the administration of allopurinol to prevent the production of superoxide anion by the action of xanthine oxidase was able to block the stimulating action of ROS on antioxidant defenses after intense exercise, such as a marathon [51]. Thus, the self-reported higher physical activity lead to increased levels of antioxidant enzymes in PBMCs, making it possible for these enzymes to be activated to cope with a subsequent exercise or an increase in ROS production and allowing a better antioxidant response.

MitND5 is a subunit of NADH dehydrogenase (ubiquinone), which is located in the mitochondrial inner membrane, and it can be considered a structural or constitutive mitochondrial protein. MitND5 did not exhibit significant changes in PBMCs between active, intermediate and inactive groups, likely suggesting that mitochondrial reticulum does not need to be more developed in elderly active men and women than in inactive participants. Fusion proteins mitofusins1 and -2 did not exhibit significant changes, while COXIV expression significantly increased five- and four-fold in active women and men, respectively, compared to the control group (inactive women). This might be due to neither a larger mitochondrial reticulum (in accordance with changeless MitND5 mRNA expression) nor enhanced biogenesis (constant Mtf1/2 mRNA expression), but caused by an enhanced respiratory/oxidative capacity of the pre-existent stock of mitochondria. This may suggest improved mitochondrial status in active subjects, but also that an active lifestyle is not enough stimulus to promote mitochondrial biogenesis in elderly active people as well as training does. Similar results were reported in elderly men and women performing resistance exercise training for 14 weeks, with a significant increase in complex IV, but without changes in mtDNA [59]. It is well established that elderly people show reduced mitochondrial content, as well as a lower mitochondrial oxidative capacity [60], but our results may point out that exercise practiced on a regular basis may slow down this decline of mitochondrial function. The present results obtained in PBMC mitochondria might differ in muscle mitochondria. In physiological conditions, peripheral tissues contain resident macrophages and dendritic cells, but other leukocytes (monocytes, neutrophils, and lymphocytes) can transiently infiltrate tissues during pathological situations. Immune cells infiltrate into skeletal muscle during contraction and injury [61]. Muscle cells (myoblasts and myofibers) may produce some of the chemoattracting and activating factors, but other resident cell types such as tissue macrophages are probably more dominant generators of chemokines and inflammatory and growth factors [62], possibly indicating different patterns and pathways modulated in muscle mitochondria.

Widespread physical inactivity is a major public health problem and improving physical activity levels is crucial for healthy aging with positive effects on energy balance and body composition. Physical activity also exerts beneficial effects against a wide range of diseases improving cardiorespiratory and muscular fitness, bone and functional health, and reducing the risk of non-communicable diseases, depression and cognitive decline [63]. Inactive, intermediate and active participants in our study reported over 2600, 5000 and 8500 METS, respectively, surpassing the general weekly recommendations to get beneficial effects on health. It is well known that regular exercise can ameliorate the oxidant–antioxidant imbalance induced by aging but it remains to be determined what precise dosage (frequency and intensity) of exercise is beneficial [64]. Overall, across all the age ranges, the benefits of implementing the above recommendations, and of being physically active, outweigh the harms.

## 5. Conclusions

To sum up, elderly people with a more active lifestyle exhibited increased available antioxidant machinery and an attenuated age-associated inflammatory status in PBCMs. Regular daily activities and an active lifestyle leads to a scenario in which the antioxidant defenses are ready to cope with subsequent stressors, allowing a better antioxidant response. No increases in mitochondrial mass or dynamics machinery is observed in subjects with active routine, but an active everyday life in healthy elderly enhances oxidative metabolism capabilities in PBMCs and the antioxidant defenses, but does not necessarily generate significant increases in oxidative stress markers in plasma. Early management of body composition and an active lifestyle could improve and maintain physical function in older adults and promote healthy aging.

## Figures and Tables

**Table 1:** Primer sequence and conditions used in Real-Time PCRs.

Gene	Primer		Temp. of Annealing
PGC-1α	Fw:	5′-CACTTACAAGCCAAACCAACAACT-3′	60 °C
	Rv:	5′-CAATAGTCTTGTTCTCAAATGGGGA-3′	
COXIV	Fw:	5′-AGAAGCACTATGTGTACGGCCC-3′	63 °C
	Rv:	5′-GGTTCACCTTCATGTCCAGCAT-3′	
MitND5	Fw:	5′-CGGCTGAGAGGGCGTAGG-3′	63 °C
	Rv:	5′-GATGAAACCGATATCCGGCCGA-3′	
Mtf1	Fw:	5′-TGT TTT GGT CGC AAA CTC TG-3′	60 °C
	Rv:	5′-CTG TCT GCG TAC GTC TTC CA-3′	
Mtf2	Fw:	5′-ATG CAT CCC CAC TTA AGC AC-3′	60 °C
	Rv:	5′-CCA GAG GGC AGA ACT TTG TC-3′	

Fw: Forward; Rv: Reverse; PGC-1α, Peroxisome Proliferator-Activated Receptor Gamma Coactivator; COXIV, cytochrome c oxidase subunit IV; MitND5, Mitochondrial NADH Dehydrogenase Subunit 5; Mtf 1, Mitofusin 1; Mtf2, mitofusin 2.

**Table 2:** Characteristics of the participants.

	Women			Men			
	(*n* = 66)			(*n* = 61)			
	Inactive	Intermediate	Active	Inactive	Intermediate	Active	
	(*n* = 20)	(*n* = 26)	(*n* = 20)	(*n* = 20)	(*n* = 15)	(*n* = 26)	ANOVA
Age (years)	67.2 ± 1.1	67.0 ± 0.8	68.1 ± 0.9	66.0 ± 1.1	66.9 ± 1.3	64.8 ± 1.1 $	G
Weight (kg)	68.6 ± 1.1	64.8 ± 0.8	64.1 ± 0.9	83.4 ± 1.1 $	80.4 ± 1.3 $	77.2 ± 1.1*$	G, E
Height (cm)	157.1 ± 1.1	156.9 ± 0.9	155.3 ± 1.1	170.5 ± 1.3 $	168.6 ± 1.4 $	169.4 ± 1.3 $	G
BMI (kg/m^2^)	27.8 ± 0.7	26.4 ± 0.8	26.6 ± 0.9	28.6 ± 0.6	28.2 ± 0.6	26.8 ± 0.6 *	E
Body fat (%)	27.0 ± 1.0	23.9 ± 1.0 *	23.1 ± 1.2 *	22.9 ± 0.9 $	22.7 ± 1.1	19.2 ± 1.3 *$#	G, E
MET min/week	2764 ± 183	5121 ± 125 *	8234 ± 437 *#	2605 ± 236	5064 ± 297 *	9135 ± 567 *#	E

Statistics: Two-way ANOVA. Results are presented as Mean ± SEM, *p* < 0.05. E indicates significant effects of Exercise. G indicates significant effects of Gender. * indicates significant differences between Active/Intermediate and Inactive groups. # indicates significant differences between Intermediate and Active groups. $ indicates significant differences between men and women.

**Table 3:** Energy and nutrient intake of the participants in the study.

	Women			Men			
	(*n* = 66)			(*n* = 61)			
	Inactive	Intermediate	Active	Inactive	Intermediate	Active	
	(*n* = 20)	(*n* = 26)	(*n* = 20)	(*n* = 20)	(*n* = 15)	(*n* = 26)	ANOVA
Energy (Kcal)	1810.2 ± 108	1595.7 ± 77	1734.5 ± 107	1754.2 ± 158	1675.9 ± 106	1605.9 ± 68	
Water (mL)	2057.6 ± 123	2023.6 ± 130	1891.8 ± 109	2053.8 ± 157	2025.4 ± 144	2134.6 ± 150	
Proteins (%)	16.9 ± 0.8	16.8 ± 0.6	17.2 ± 1	17.4 ± 1	19.3 ± 0.8	18.2 ± 0.9	
Carbohydrates (%)	43.3 ± 2	45.2 ± 1	44.8 ± 2.1	46.0 ± 2	44.6 ± 2	41.9 ± 2	
Lipids (%)	35.7 ± 2	33.9 ± 1	34.6 ± 2	33.5 ± 2	33.2 ± 2	35.8 ± 1	
Fiber (%)	3.0 ± 1	3.2 ± 0.2	2.9 ± 0.3	3.5 ± 0.4	3.1 ± 0.2	3.2 ± 0.3	
Vitamin E (mg/day)	8.2 ± 0.8	7.5 ± 0.7	8.3 ± 1	8.6 ± 0.9	11.4 ± 1	8.4 ± 1	
Vitamin C (mg/day)	130 ± 17	140 ± 14	171 ± 17	131 ± 17	134 ± 17	137 ± 19	
Selenium (µg/day)	77.5 ± 11	75.7 ± 9	87,9 ± 6	136 ± 6 $	112 ± 11 $	124 ± 11 $	G
Zinc (mg/day)	7.6 ± 0.5	7.5 ± 0.6	8.1 ± 0.5	9.7 ± 0.6 $	9.5 ± 0.8 $	9.2 ± 0.6 $	G

Statistics: Mean ± SEM, *p* < 0.05. Two-way ANOVA. G indicates significant effects of Gender. $ indicates significant differences between men and women.

**Table 4:** PBMC blood count.

	Women			Men			
	(*n* = 66)			(*n* = 61)			
	Inactive	Intermediate	Active	Inactive	Intermediate	Active	
	(*n* = 20)	(*n* = 26)	(*n* = 20)	(*n* = 20)	(*n* = 15)	(*n* = 26)	ANOVA
PBMCs (10^3^ cells/mm^3^)	2.13 ± 0.13	1.90 ± 0.19	1.65 ± 0.19 *	1.99 ± 0.19	1.91 ± 0.2	1.33 ± 0.2 *	E
Lymphocytes (%)	33.2 ± 1.5	33.5 ± 1.3	32 ± 1.2	34 ± 1.2	33 ± 1.6	32 ± 1.1	
Monocytes (%)	6.6 ± 0.4	7.3 ± 0.4	6.5 ± 0.3	7.3 ± 0.4	7.6 ± 0.4	6.6 ± 0.3	

Statistics: Two-way ANOVA. Results are presented as Mean ± SEM, *p* < 0.05. E indicates significant effects of Exercise. * indicates significant differences between Active/Intermediate and Inactive groups.

**Table 5:** Peripheral blood mononuclear cells (PBMCs) protein levels.

		Inactive			Intermediate			Active			ANOVA		
											G	E	SxE
CAT (%)	Women	100	±	9	114	±	13	146	±	17 #	0.001	0.013	0.601
	Men	132	±	12	170	±	17 *	175	±	17 #$			
MnSOD (%)	Women	100	±	10	116	±	13	156	±	21 #	0.420	0.045	0.900
	Men	125	±	13	127	±	20	162	±	24			
GRd (%)	Women	100	±	10	109	±	10	165	±	20 #	0.013	0.000	0.759
	Men	123	±	20	158	±	21	209	±	24 #$			
GPx (%)	Women	100	±	12	111	±	14	168	±	26 #	0.994	0.002	0.720
	Men	108	±	9	120	±	17	152	±	21 #			
TrxR1 (%)	Women	100	±	13	135	±	16	172	±	24 #	0.056	0.001	0.853
	Men	124	±	24	177	±	23 *	196	±	21 #			
UCP3 (%)	Women	100	±	10	102	±	9	118	±	15	0.099	0.039	0.565
	Men	112	±	10	109	±	8	152	±	19 #			

Statistics: Two-way ANOVA. Results are presented as Mean ± SEM, *p* < 0.05. E indicates significant effects of Exercise. G indicates significant effects of Gender. * indicates significant differences between Active/Intermediate and Inactive groups. # indicates significant differences between Intermediate and Active groups. $ indicates significant differences between men and women.

**Table 6:** Relative mRNA expression in PBMCs.

	Women			Men			
	(*n* = 66)			(*n* = 61)			
mRNA levels	Inactive	Intermediate	Active	Inactive	Intermediate	Active	
(%)	(*n* = 20)	(*n* = 26)	(*n* = 20)	(*n* = 20)	(*n* = 15)	(*n* = 26)	ANOVA
COXIV	1 ± 0.4	1.39 ± 0.5	5.25 ± 1.9 *	1.23 ± 0.4	1.53 ± 0.8	4.37 ± 1.9 *	E
PGC1α	1 ± 0.9	1.11 ± 0.7	1.91 ± 1.0	1.08 ± 0.9	1.12 ± 0.4	1.19 ± 0.6	
MitND5	1 ± 0.4	0.96 ± 0.5	0.72 ± 0.3	1.32 ± 0.5	0.57 ± 0.01	1.05 ± 0.4	
Mtf1	1 ± 0.3	0.80 ± 0.2	1.55 ± 0.8	0.79 ± 0.3	1.48 ± 0.7	1.38 ± 0.5	
Mtf2	1 ± 0.5	1.02 ± 0.7	1.91 ± 0.8	0.83 ± 0.3	1.13 ± 0.7	1.49 ± 0.6	

Statistics: Two-way ANOVA. E indicates significant effects of Exercise. G indicates significant effects of Gender. Results are presented as Mean ± SEM, *p* < 0.05. * indicates significant differences between Active/Intermediate and Inactive groups.

**Table 7:** Enzymatic activities in PBMCs and plasma.

		Women			Men		
		(*n* = 66)			(*n* = 61)		
		Inactive	Intermediate	Active	Inactive	Intermediate	Active
		(*n* = 20)	(*n* = 26)	(*n* = 20)	(*n* = 20)	(*n* = 15)	(*n* = 26)
PBMCs	CAT (K/10^9^ cells)	53 ± 24	44 ± 25	32 ± 13	38 ± 15	36 ± 18	71 ± 30
	SOD (pkat/10^9^ cells)	57 ± 16	84 ± 27	110 ± 31	71 ± 39	69 ± 27	64 ± 16
	GPx (nkat/10^9^ cells)	102 ± 31	77 ± 14	89 ± 21	80 ± 29	95 ± 38	54 ± 19
	GRd (nkat/10^9^ cells)	447 ± 174	406 ± 102	420 ± 166	326 ± 100	324 ± 67	303 ± 84
Plasma	CAT (K/L)	43 ± 20	51 ± 15	54 ± 17	41 ± 17	44 ± 14	64 ± 14
	SOD (pkat/L)	736 ± 117	536 ± 72	526 ± 76	724 ± 108	678 ± 103	649 ± 67

Statistics: Two-way ANOVA. No significant changes between genders were found, and no effects of physical activity were detected.
